# Literary Gossip and Record

**Published:** 1862-10

**Authors:** 


### Art. XI.—LITERARY GOSSIP AND RECORD.

In our last number we gave a brief account of certain observations of M. Chabas, in his lately published "Melanges Egyptologiques,"* on the antiquity of plague. In the same work some curious information is found on the medicine of the ancient Egyptians and the antiquity of clysters, derived from an unpublished hieratic papyrus, known as the medical papyrus, and preserved in the museum of Berlin. This work contains not less than one hundred and seventy medical prescriptions applicable to a very great number of maladies. Among these are poultices, liniments, draughts, forms of diet, &c. For example :—

"Remedy which quickly cures internal tumours : vegetable *auau*, white corn, pinch of *ar*, pinch of natural nitre, corn bran ; mix together, and apply externally.

"Remedy which destroys pimples : equal parts of beef and honey ; eat during four days.

"Remedy which destroys pimples in infants : white corn bran . . . ; mix with milk, and give the infant to drink."

There are also given topical applications for bruises, the bites of vermin, cuts, burns, &c :—

"Liniment which destroys burns : herb *tahaa* pounded in honey ; anoint the patient with it.

"Another unguent : *bak*, common salt . . . . incense ; mix together.

"Remedy which relieves painful twitches in spring and winter in all the members : stag's horn beaten in sweet *hak* (wine probably), grain *auau*," &c.

The formulæ are, for the most part, of the greatest simplicity.

Certain hints are also given on the diagnosis of several diseases. The malady to which the greatest attention is accorded would appear to have its seat in the intestines, but it also attacks the members. M. Chabas thinks that inflammation of the intestines is referred to, accompanied with feeling of weight, hardness of the abdomen, and tenesmus, and swelling of the articulations.

"His belly is heavy," says the papyrus, "he has sickness and burning of the stomach . . . , he is sluggish and chilly, thirsty in the night, his taste is perverted, as if he had eaten sycamore figs, his flesh is deadened ; if he goes to stool, his bowels refuse to act. Say . . . . that there is a nidus of inflammation in the belly," &c.

Several curious anatomical details are given descriptive of the vehicles of vital and morbid principles :—

---

* Chalon-sur-Saone, 1862.

"The head of man has thirty-two vessels; they carry breath to his heart, and give breath to all his members." "There are two vessels in the breast; they give warmth to the lungs," &c. "There are two vessels in the legs. If there is pain in the legs, and the arms are powerless, it is because the secret vessel of the leg is affected." "There are two vessels in the arms. If an arm becomes disordered, if there are twitches in the fingers, say that it is a case of twitches," &c. "There are two vessels of the occiput, two of the sinciput, two of the interior, two of the eyelids, two of the nostrils, and of the left ear. The life-breath enters by them."

After a rubric in which dilatation of the heart (stomach) is in question, and of the lost functions of digestion, the text adds:—

"There is disorder of the fundament: it is not possible to stool. This is brought about, even to a fatal ending, by the vessels of the feet."

Does the word which M. Chabas translates vessels, refer to the arteries, the veins, or the nerves? he asks, or simply to imaginary conduits? In the papyrus of Leyden we read:—

"Open your mouths, O vessels of such a one, son of such a one; reject the malady which is in you! I address myself to you, you vessels which have received the evils," &c.

Be this as it may, this disorder of the intestines, produced by the vessel of the foot, was considered a very serious malady, and a profuseness of medication truly formidable is directed fo its relief. As a sample: it is prescribed that at first cow's milk should be taken, boiled, mixed with a liquid named *ar*, then strained; next honey is to be added, and the preparation thus made is to be used as a drink four days; after this, goat's milk with honey is to be made use of in the same manner. Clysters are also prescribed, composed of the "*serum* of human milk, to be administered at daybreak;" or of honey, common salt, and three or four unknown ingredients, in various combinations. sometimes with the addition of oil. The clyster is denominated *a potion for* or *by the fundament.*

We know that the use of clysters is of great antiquity. Diodorus of Sicily and Pliny speak of this therapeutical means employed by the Egyptians. Tradition attributes the invention to the ibis. According to Pliny, this bird injects water into its intestines through the fundament, by means of its beak. This ridiculous fable has arisen from the misinterpretation of an Egyptian symbol. The papyrus contains twenty-eight receipts for clysters, prescribed for intestinal pains, sanguinolent stools and urine, pain in micturition, abnormal urine, and a disease of the bone.

The papyrus also gives several very curious directions for ascertaining the existence or possibility of pregnancy, and the sex of the fœtus.

M. Chabas terminates his valuable critical examination of this papyrus with an account of the different materials used in the preparation of the various remedies.

*Honey* occurs in the majority of the potions. Dioscorides speaks of its employment for internal maladies; and Galen for clysters. *Wine* is used mingled with honey, incense, blood, &c. Similar mixtures are named by Dioscorides and Galen. *Milk*, human, and of the goat and the cow; also milk of woman confined of a boy. Pliny speaks of the special use of this milk; and Galen and Dioscorides of the employment of milk variously mingled, as directed in the papyrus, especially with honey and oil, and of the use for clysters. *Oil; hak,* a fermented liquor; sweet *hak; bak,* a liquor furnished by the tree *bak,* the character of which is doubtful; *salt; nitre* or *sal ammoniac; incense;* the *blood* and *flesh of animals; stag's horn, urine,* &c.; *lapis memphites;* a cupreous and other minerals, and *sawdust of cedar.* Such are the chief medicaments mentioned in the medical papyrus, and the majority of which and chief uses by the ancients are already familiar through the writings of Dioscorides and Galen.

